# Harnessing Ratoon Cropping for Mitigating Greenhouse Gas Emissions

**DOI:** 10.3390/biology15191751

**Published:** 2026-10-02

**Authors:** Xin Zhang, Hai Huang, Zengqiang Li, Xiangjun Kong, Aziz Khan, Yanbo Zhang

**Affiliations:** 1 Xinxiang Key Laboratory of Crop Root Biology and Green Efficient Production, Henan Key Laboratory of Quality and Stress-Resilient Biobreeding and Safe Production of Cotton and Oil Crops, Henan Institute of Science and Technology, Xinxiang 453003, China; xinzhang@hist.edu.cn (X.Z.);; 2 College of Agriculture, Guangxi University, Nanning 530005, China; 3 Xianyang Agricultural Science Research Institute, Xianyang 712099, China

**Keywords:** agroecosystems, global warming potential (GWP), greenhouse gas intensity (GHGI), no-tillage, ratoon rice, soil organic carbon sequestration, alternate wetting and drying (AWD), enhanced efficiency fertilizers

## Abstract

Ratoon cropping systems have gained increasing attention for their potential to mitigate greenhouse gas (GHG) emissions, including carbon dioxide (CO_2_), methane (CH_4_), and nitrous oxide (N_2_O). Building on the well-established root system of the previous crop, ratoon crops require no land preparation, sowing, or transplanting and need less fertilizer, water, and pesticides than annual crops while achieving earlier harvests, thereby reducing GHG emission intensity (GHGI) by 7.9–17.3% and N_2_O emissions by 40.4–78.1%. Reduced fertilization, efficient water management, and integrated pest management can further curtail GHG emissions, while improved soil fertility, sustainable land management, and cover crop integration enhance soil carbon sequestration. Genetic improvement of ratoon crops can additionally enhance stress tolerance and resource use efficiency, reducing fertilizer requirements and ultimately lowering GHG emissions. This review also addresses ratoon cropping in dryland systems, including sugarcane, sorghum, cotton, and pigeon pea, highlighting their distinct GHG emission profiles and mitigation potential. Its innovative contribution is an integrated framework—synthesizing crop physiology, soil carbon processes, agronomic management, and genetic improvement—that evaluates GHG mitigation opportunities across both ratoon rice and dryland ratoon crops while explicitly addressing trade-offs, context-dependency, and evidence gaps.

## Simple Summary

Ratoon cropping systems—which reuse existing root systems to skip land preparation, sowing, and transplanting—can significantly reduce greenhouse gas emissions (CO_2_, CH_4_, N_2_O) by requiring less fertilizer, water, and pesticides. Further emission reductions are achieved through optimized nitrogen use, efficient water management, integrated pest control, enhanced soil carbon sequestration, and genetic improvements that boost stress tolerance and resource efficiency.

## 1. Introduction

Agricultural production is a significant source of anthropogenic greenhouse gas (GHG) emissions, accounting for approximately 10–12% of the global total [1,2]. Emissions of carbon dioxide (CO_2_) arise primarily from energy-intensive farm operations and input manufacturing [3], while methane (CH_4_) and nitrous oxide (N_2_O), with global warming potentials approximately 27.9 and 273 times that of CO_2_ over a 100-year time horizon (IPCC Sixth Assessment Report, AR6), respectively, are particularly potent greenhouse gases [4]. This underscores the critical need for innovative agricultural practices that can decouple food production from its climate impact.

**Figure 1:**
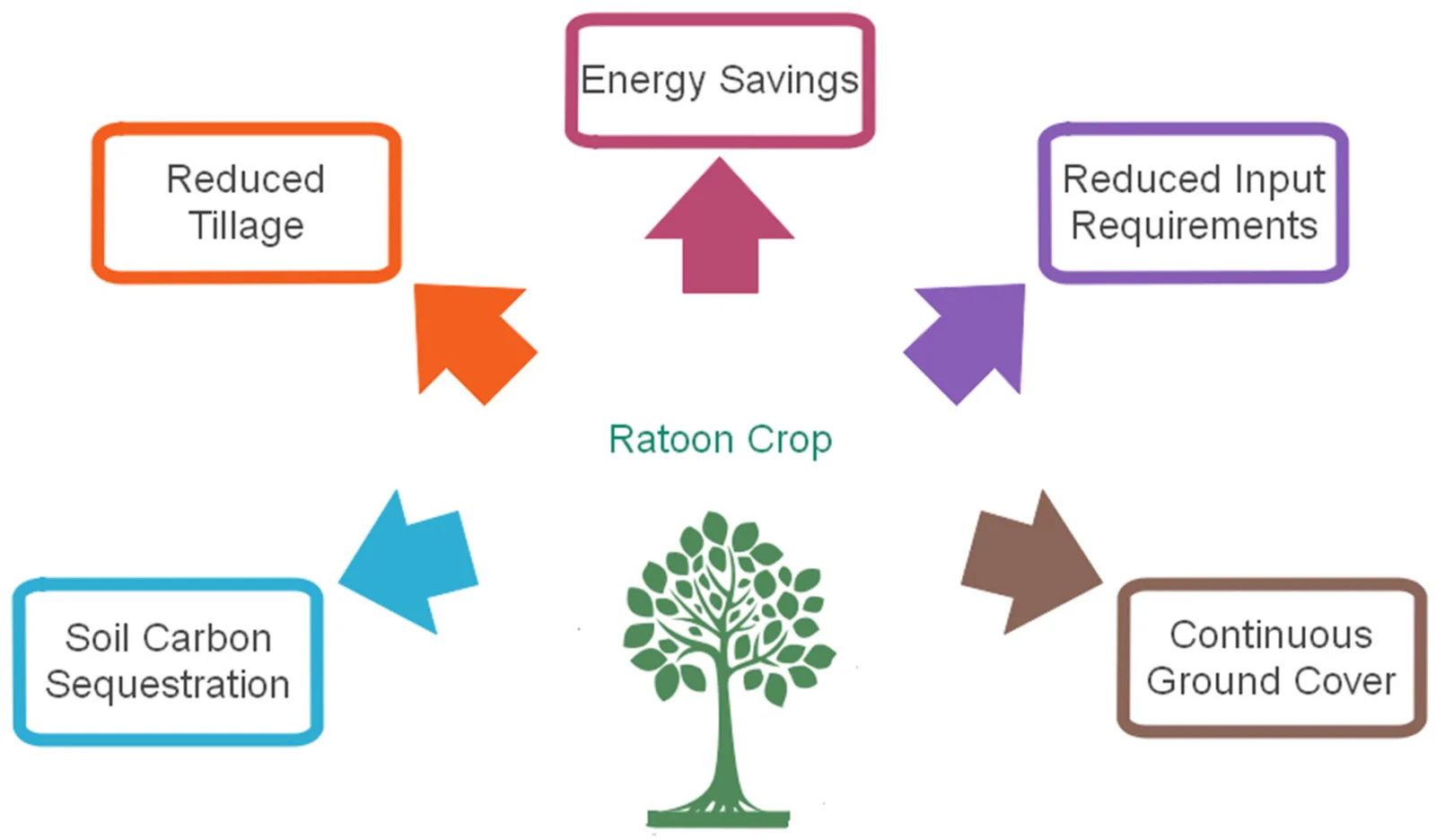
Five special advantages of ratoon cropping in reducing GHG emissions.

Ratoon cropping (stubble/regenerative cropping) – cultivating crops from residual stubble/roots instead of annual replanting – originated in tropical/subtropical regions and has been applied to traditional crops (sugarcane, rice, sorghum) and expanded to maize, cotton, and wheat with agricultural advancements. However, the global expansion of ratoon cropping to cool-season cereals such as wheat faces physiological and commercial constraints. Wheat, as a cool-season cereal, requires a vernalization period, specific photoperiod responses, and heat stress that are not easily accommodated by ratoon management in many temperate regions. Similarly, warm-season cereals may face cold stress during the ratoon regrowth phase in increasingly variable climates. These constraints underscore the need for crop-specific assessments of ratoon feasibility. GHG mitigation advantages of ratoon cropping (Figure 1) include: (1) enhanced soil carbon sequestration: decomposing residues of previous crops increase soil organic carbon, offsetting atmospheric CO_2_ [5]; (2) reduced tillage and energy savings: intact stubble/roots enable minimal/no tillage (preserving soil structure and organic matter), while eliminating land preparation/planting reduces machinery fuel consumption and associated emissions [6,7]; (3) lower input requirements: residue decomposition releases nutrients, reducing synthetic fertilizer/pesticide use; surface residues suppress weeds, minimizing herbicide needs [8]; (4) continuous ground cover: it prevents soil erosion/runoff, preserving organic matter and soil fertility [9].

While previous reviews and studies [4,5,6,10,11] have focused on singular aspects of ratoon cropping, such as its agronomic yield potential or its impact on a specific GHG (e.g., CH_4_ in rice systems), this review synthesizes and analyzes the system’s multi-faceted role in comprehensive GHG mitigation. The novel contribution of this work lies in its integrated framework that connects agronomic management, environmental factors, and systematic processes to the overarching goal of reducing the carbon footprint of agriculture.

## 2. The Ratooning Process: Agronomic and Physiological Foundations

### 2.1. Ratoon Rice (Oryza sativa *L*.)

The regeneration process in rice, known as ratooning, is not merely a second harvest but a complex physiological response to the initial harvest. Upon cutting of the main crop, dormant buds (tillers) located at the basal nodes of the rice stubble are activated to develop into new, productive tillers. This process is governed by the redistribution of residual nutrients and carbohydrates from the root system and lower stubble of the main crop, which directly fuels the initial growth of the ratoon tillers [12].

#### 2.1.1. Key Growth Stage Differences: Main Crop vs. Ratoon Crop

A paramount distinction lies in the growth cycle duration. The ratoon crop bypasses the lengthy vegetative establishment phase required from seed. Empirical data from controlled field experiments consistently demonstrate a significant shortening of the growth period. The ratoon crop’s cycle is typically 35–60% shorter than that of the main crop [13,14]. For instance, where a main rice crop may require 151 days to mature, subsequent (first, second, and third) ratoon crops can mature in 81, 126, and 80 days, respectively [15]. 

#### 2.1.2. Plant Height Comparison: Main Crop vs. Ratoon Crop

Ratoon rice (RR) plants are consistently and significantly shorter than main crop plants. This is a well-documented phenomenon attributed to the compressed growth cycle and the developmental priority shift towards rapid panicle formation rather than extensive stem elongation. Quantitative analyses demonstrate a substantial reduction in plant height for ratoon crops, with decreases ranging from 24.4–84.4% compared to the main crop [12], as exemplified by field measurements documenting a decline from 96 cm in the main crop to 33.4 cm in the ratoon crop [14].

#### 2.1.3. Tiller Number and Dynamics: Main Crop vs. Ratoon Crop

The tillering dynamics represent a more complex and variable trait. While the main crop develops tillers from the seed, the ratoon crop generates tillers from basal buds. The number of productive (effective) tillers per hill is generally lower in the ratoon crop than in the main crop [10]. The yield of the ratoon crop is directly dependent on the number of regenerative tillers that successfully develop [12]. However, some genotypes and under optimal management, the ratoon crop can produce a higher total number of tillers by 19% [16], though a significant portion of these may be unproductive. The number of tillers in the main crop is a strong predictor for ratoon crop success, as a robust main crop supports a higher number of dormant buds [17]. 

#### 2.1.4. Visual Appearance and Other Morphological Traits

Beyond height and tillering, the visual appearance differs. RR (ratoon rice) often exhibits a more rapid canopy closure and a different growth architecture due to its simultaneous vegetative and reproductive growth. Studies utilizing high-throughput phenotyping and spectral analysis (e.g., NDVI) have begun to model these differences to predict RR growth and yield based on the main crop’s characteristics [18]. Furthermore, a meta-analysis revealed that grain quality was improved in the ratoon crop compared with the main crop [19].

### 2.2. Other Major Ratoon Crops

The physiological basis of ratooning varies across species, but the common principle is the regeneration from pre-existing meristematic tissues. Here we list four other ratoon crops.

#### 2.2.1. Sugarcane (*Saccharum* spp.)

Sugarcane is a classical ratoon crop, typically harvested multiple times (plant crop, first ratoon, second ratoon) before replanting. Sugarcane regenerates from buds left on the stubble after harvest. A quantitative analysis of successive ratoon cycles revealed a genotype-dependent reduction in stalk diameter by 4.86% in the first ratoon and 2.84% in the second ratoon compared to the plant cane, whereas average stalk length exhibited a modest increase of 1.92% and 1.28%, respectively [20]. Yields and sucrose contents of subsequent ratoon crops are usually lower than those of the plant crop. This decline is not universal; selective breeding can produce clones that maintain or even increase yield and sucrose content in ratoon cycles [21]. 

#### 2.2.2. Sorghum (*Sorghum* spp.)

Sorghum’s robust tillering ability makes it highly suitable for ratooning, a practice used for both grain and forage production. After the main crop is harvested, regrowth initiates from buds at the basal nodes of the sorghum stubble. Ratoon sorghum cultivation shortens the crop life cycle by approximately 25 days and enhances the production of both fresh and dry green forage due to its elevated tillering capacity compared to direct seeding methods [22]. Additionally, ratoon sorghum exhibits strong competitive traits against weeds. The success of ratoon sorghum is contingent on the height of the cut, the viability of the buds, and the residual moisture and nutrients in the soil. A key factor for high ratoon yield is promoting the development of vigorous tillers with large stem diameters, as these are directly correlated with grain yield in the ratoon crop [23].

#### 2.2.3. Cotton (*Gossypium* spp.)

Ratoon cotton, while not a common practice, has the perennial potential to regrow as a multi-branched shrub from the woody stem in frost-free regions, a starkly different architecture from a seed-grown plant. Ratoon cotton initiates flowering 4–6 weeks earlier than seed-grown counterparts in the next year due to pre-established root systems that bypass juvenile growth stages, thereby extending the reproductive period and enhancing yield potential [24]. 

#### 2.2.4. Pigeon Pea (*Cajanus cajan*)

Pigeon pea is a botanically perennial legume with a strong natural capacity for ratooning. Under favorable conditions (adequate soil moisture, temperature), the pigeon pea plant will regenerate new shoots from its perennial stubble after the main harvest [25]. Remarkably, and in contrast to sugarcane, ratoon crops of pigeon pea can outperform the initial crop. This is attributed to a more established root system and a change in growth habit [25]. Pod set on ratooned plants is often higher due to greater flower retention [26]. The success of ratooning is highly genotype-dependent and influenced by maturity group and environmental factors [27].

## 3. GHG Emissions in the Ratoon Cropping System

### 3.1. GHG Emissions in the RR Cropping System

Paddy fields are an essential source of GHG emissions in agriculture [28]. Globally, rice accounts for 22% and ~11% of total agricultural CH_4_ and N_2_O emissions, respectively [11]. Changes in cropping systems can inevitably lead to changes in GHG emissions. The CH_4_ emissions from straw residue decomposition during the ratoon season are higher than those of the main crop [29], but significantly lower than those of ordinary double-season rice (DR) [30]. However, when expressed as yield-scaled GHG intensity (GHGI), the ratoon system can show advantages. The significant reduction in CH_4_ emissions during the ratoon season alone is due to low temperature, short growth period, and low aboveground biomass [4]. Moreover, the emissions of the GHGs (CO_2_, N_2_O, CH_4_) from rice ratoon cropping systems are significantly reduced compared with single cropping systems when producing the same amount of protein [30,31].

#### 3.1.1. Mechanistic Insights into GHG Mitigation in Ratoon Rice

The unique agronomic characteristics of the RR system—specifically the carry-over of a mature, living root system and the decomposition of crop residues in an anaerobic environment—are central to its GHG emission profile. Understanding the fundamental mechanistic and microbial processes is the first step toward effective mitigation.

##### CH_4_ Emission Mechanism in Ratoon Rice

RR systems amplify CH_4_ emissions via anaerobic decomposition of residual roots/stubble (carbon sources) by methanogenic archaea [32]. Methanogenesis follows two key pathways: hydrogenotrophic (CO_2_ + 4H_2_ → CH_4_ + 2H_2_O) and acetoclastic (CH_3_COOH → CH_4_ + CO_2_). The *Mcr* gene (marker for methanogenic activity) and *pmoA* gene (marker for methanotrophic bacteria that oxidize CH_4_ to CO_2_) regulate net CH_4_ flux, balancing production (anaerobic bulk soil) and oxidation (aerobic root/soil-water interfaces) [33].

##### N_2_O Production and Enzymatic Controls

N_2_O is a byproduct of several microbial nitrogen transformation processes, including (1) nitrification, which is the aerobic oxidation of ammonia (NH_4_^+^) to nitrate (NO_3_^−^). Under oxygen-limiting conditions, N_2_O can be produced as a byproduct (2NH_4_^+^ + 3O_2_ → 2NO_2_^−^ + 4H^+^ + 2H_2_O; 2NO_2_^−^ + 2H^+^ → N_2_O + H_2_O + O_2_). The first, rate-limiting step is catalyzed by ammonia monooxygenase (AMO); (2) denitrification is the anaerobic respiration process where nitrate or nitrite is used as an electron acceptor, leading to the sequential reduction to N_2_ gas (2NO_3_^−^ → 2NO_2_^−^ → 2NO → N_2_O → N_2_). When this process is incomplete, N_2_O is a major end-product that escapes to the atmosphere; (3) nitrifier denitrification: the direct reduction of nitrite to N_2_O by ammonia-oxidizing bacteria (NH_2_OH + NO_2_^−^ + H^+^ → N_2_O + 2H_2_O); (4) chemodenitrification: abiotic reactions, particularly in acidic soils with high concentrations of reduced iron (2NO_2_^−^ + 4Fe^2^^+^ + 6H^+^ → N_2_O + 4Fe^3^^+^ + 3H_2_O).

The enzymatic control of these pathways is critical. Denitrification is catalyzed by a series of reductases: nitrate reductase (NR, encoded by *nar* genes), nitrite reductase (NiR, *nir* genes), nitric oxide reductase (NOR, *nor* genes), and nitrous oxide reductase (N_2_O reductase, *nosZ* gene) [34]. The expression and activity of these enzymes are regulated by oxygen levels, pH, and the availability of carbon and nitrogen substrates. N_2_O accumulation occurs when the rate of N_2_O production exceeds its reduction to N_2_ by N_2_O reductase. Factors that inhibit *nosZ* expression or activity, such as low pH or limited bioavailable copper, can lead to a high N_2_O: N_2_ emission ratio [35].

In contrast to the increase in methane emissions, the same study reported that total N_2_O emissions from the RR system were 40.4–78.1% lower than those from the main season [36]. This reduction was attributed to two primary mechanisms. First, the ratoon crop typically requires less nitrogen fertilizer than a newly planted main crop, thus reducing the available nitrogen substrate for N_2_O production via nitrification and denitrification. Second, the absence of tillage and the maintenance of continuously flooded, strongly anaerobic conditions in the ratoon system may promote complete denitrification of nitrates to inert dinitrogen gas (N_2_) rather than the intermediate product, N_2_O [31]. It is important to note that data presented in this section encompass three distinct measurement frameworks: (i) the main crop season alone, (ii) the isolated ratoon season alone, and (iii) the cumulative integrated system (main crop + ratoon crop from a single soil preparation event); these should not be conflated, as they address different questions about system performance. In main-crop paddies, water-saving practices such as AWD have been shown to decrease the abundance and transcription of *mcrA* while increasing relative *pmoA* activity, at the cost of enhanced N_2_O pulses associated with *nirK*/*nirS*-mediated denitrification [37,38]. However, direct molecular evidence linking such management-induced shifts in root-zone gene expression to integrated GHG fluxes is lacking for the ratoon season specifically. Future research should therefore prioritize paired measurements of root-zone gene expression (*mcrA*, *pmoA*, *amoA*, *narG*, *nirK*/*nirS*, *norB*, *nosZ*) and chamber-based fluxes across both the main and ratoon seasons of RR systems.

##### Carbon Sequestration and Root–Soil–Microbe Interactions

While RR systems are a source of GHGs, they also possess a significant, and often overlooked, potential for soil organic carbon (SOC) sequestration. Carbon sequestration in this context is the net accumulation of carbon in the soil, derived primarily from plant biomass (roots, stubble, exudates) that is not fully respired back to the atmosphere as CO_2_ or CH_4_. The key to this potential lies in the continuous living root system. Unlike conventional single-harvest systems where the field lies fallow, the ratoon system maintains an active rhizosphere for an extended period. This continuous input of root exudates and fine root turnover facilitates microbial activity that is essential for the formation and stabilization of soil aggregates [39]. These aggregates physically protect organic matter from decomposition, trapping it within their structures and effectively sequestering it for the medium term [40,41]. Research has also highlighted the “rhizosphere microecological mechanism of carbon sequestration” in rice ratooning, suggesting that the unique interactions at the root–soil–microbe interface are central to this process [42]. It should be emphasized, however, that aggregation, root inputs and rhizosphere community shifts are proposed stabilization mechanisms; they demonstrate enhanced carbon input and short-term retention, but do not by themselves establish sustained net SOC accumulation.

Direct SOC-stock measurements in ratoon systems are scarce, and the strongest quantitative field evidence comes from multi-ratoon sugarcane. In a 6-year split-plot field experiment in semi-arid India (one plant crop followed by four ratoon crops), Pradhan et al. [5] compared residue retention with the conventional practice of residue burning, with three fertigation-based nutrient regimes as subplots. SOC stocks were determined from samples collected at the start of the experiment (baseline) and after 6 years, for the 0–30 cm depth increment, and were expressed on a per-unit-area basis for this layer; no equivalent-soil-mass correction was reported, so treatment-induced differences in bulk density cannot be fully excluded. Residue retention increased total SOC by 21% relative to burning, enriched the very labile, labile, less-labile and non-labile pools by 42, 47, 17 and 13%, respectively, and corresponded to a sequestration rate of 0.68 Mg C ha^−^^1^ yr^−^^1^ with a carbon retention efficiency of 37%; passive pools contributed 72–75% of total SOC stock, accompanied by higher enzyme activities and microbial counts [5].

For ratoon rice, repeated SOC-stock determinations are essentially lacking. The closest available evidence is a two-season field study (2023–2024, subtropical China) in which the ratoon rice system (main + ratoon crop) acted as a net carbon sink, sequestering 3.12–10.47 t CO_2_-eq ha^−^^1^, whereas the single-crop control functioned as a carbon source; the surplus was attributed to markedly reduced rhizodeposition (−72 to −77%) and an increased abundance of autotrophic bacteria (+32 to +56%) in the ratoon season [43]. Because this result derives from a net ecosystem carbon budget (NECB) rather than from repeated SOC-stock measurements, it should be interpreted as inferred, short-term net carbon retention rather than demonstrated sequestration. Overall, most evidence for SOC accumulation in ratoon systems is derived from short-term studies (<5 years) or model-based estimates; long-term field measurements (>10 years) confirming sustained net SOC accumulation are limited. The remaining evidence gap includes the need for multi-year SOC-stock measurements across diverse soil types and climate zones, with explicit reporting of sampling depth, study duration, baseline, and bulk-density correction (ideally on an equivalent-soil-mass basis), together with deeper soil layers beyond the top 30 cm and the permanence of accrued carbon under the disturbance events that punctuate successive ratoon cycles.

However, a critical trade-off exists. The very same carbon inputs that drive SOC sequestration—root exudates and decaying biomass—are also the primary substrates for methanogenesis [44]. Therefore, the net climate impact of an RR system depends on the intricate balance between carbon sequestration in the soil and carbon loss as CH_4_. This balance is mediated almost entirely by the rhizosphere microbiome [45]. To quantify this trade-off, the Net Global Warming Potential (Net GWP) framework should be employed: Net GWP = GWP_CH_4_ + GWP_N_2_O − SOC_sequestered (expressed as CO_2_-equivalents), where GWP values are calculated using the chosen IPCC assessment report coefficients. Given the qualifications above, the SOC_sequestered term should be propagated with an explicit uncertainty range (or set to zero in a conservative scenario) whenever it rests on NECB or model estimates rather than on measured stocks. Additionally, a comprehensive life-cycle assessment (LCA) should incorporate indirect gains from reduced fuel consumption in machinery operations and fertilizer savings associated with ratoon cropping, which are often omitted in soil-only emission inventories. Reported LCA comparisons indicate that the indirect gains of ratoon systems arise mainly from the elimination of land–preparation and planting operations (lower machinery fuel use) and from lower fertilizer, water, and pesticide requirements [6,7,31]; a quantitative summary table will be provided in the final version.

#### 3.1.2. Genetic Control

There is growing evidence that GHG emissions are a heritable trait in rice. Cultivars differ significantly in their methane emission potentials, a variation linked to a suite of physiological and morphological traits that are under genetic control [46,47]. Key traits include (1) root system architecture and exudation: The quantity and quality of root exudates, which are the primary fuel for methanogens, vary among genotypes [48]. Breeding for cultivars with lower exudation rates or with exudate profiles that are less favorable to methanogens is a promising mitigation strategy; (2) aerenchyma development: The gas-filled tissues that transport oxygen to the roots also serve as conduits for CH_4_ to escape the soil, bypassing microbial oxidation. Cultivars with less developed aerenchyma may exhibit lower CH_4_ flux; however, this breeding direction involves critical trade-offs. Reduced aerenchyma development can limit root oxygen supply under waterlogged conditions, potentially impairing root function, nutrient uptake, and overall ratooning vigor. Therefore, optimal breeding strategies should seek a balance between minimizing CH_4_ conductance and maintaining adequate aerenchyma for root oxygenation and plant vigor, particularly under stress conditions; (3) ratooning ability: The genetic capacity of a cultivar to regrow vigorously after the main harvest is itself a key trait. Research has identified quantitative trait loci (QTLs) associated with ratooning ability [49], which could be combined with low-emission traits through marker-assisted breeding; (4) nitrogen metabolism: The genetic regulation of enzymes like nitrate reductase (NR) within the rice plant determines its nitrogen use efficiency [50]. Plants with more efficient N uptake and assimilation may require less fertilizer, indirectly reducing the substrate available for N_2_O production by soil microbes.

#### 3.1.3. Metabolomic Influences

Metabolomics, the study of small-molecule metabolites, provides a powerful lens to understand the chemical communication between the rice plant and the GHG-producing microbes in its rhizosphere. The composition of root exudates is the primary mechanism of this communication. Metabolomic profiling using techniques like GC-MS and LC-MS has revealed that rice roots release a complex cocktail of sugars, organic acids, amino acids, and phenolics [51,52]. These metabolites directly regulate microbial activity. For instance, simple sugars and organic acids are readily metabolized by fermentative bacteria, producing the acetate and H_2_ that fuel methanogenesis [44]. Therefore, the metabolic profile of a cultivar’s exudates can directly select for a more or less methanogenic microbiome. For example, a study using a low methane-emitting rice line found that reduced root exudation was a key factor in suppressing methane emissions [53].

#### 3.1.4. Synthesis and Additional Considerations

**Figure 2:**
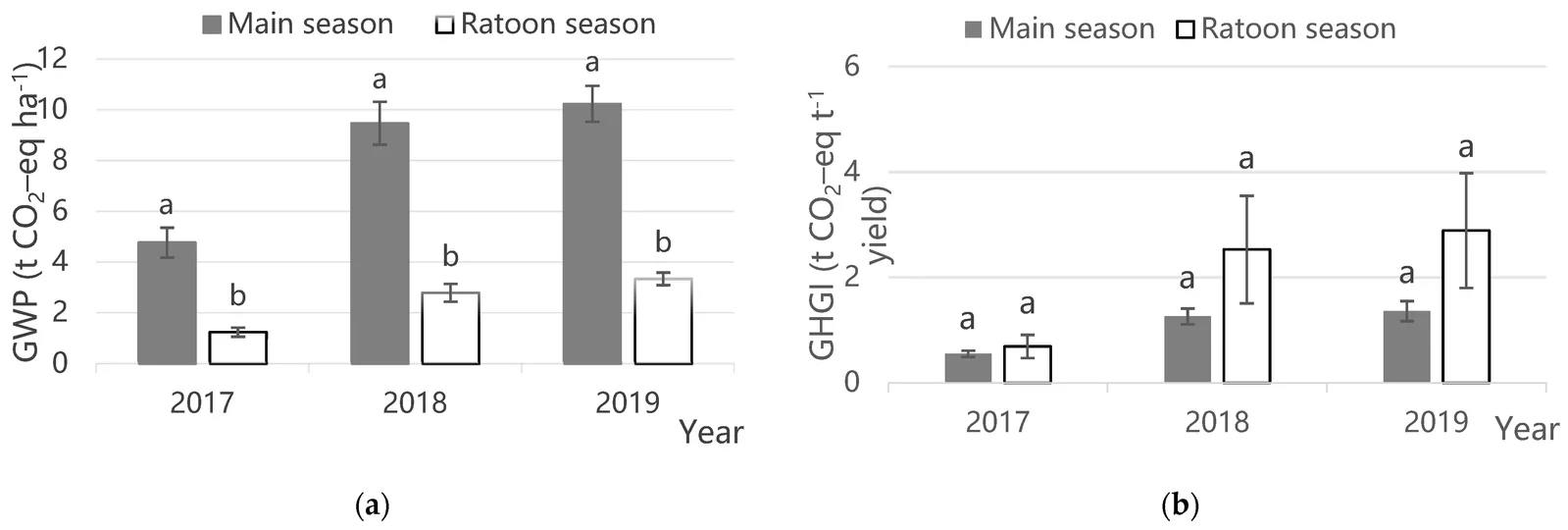
Comparisons of area-based GWP (global warming potential) (**a**) and yield−scaled GHGI (greenhouse gas intensity) (**b**) between the main season and the ratoon season of rice in different years. Note: Data in the figure are derived from the average values of five rice varieties in the same year of [54]. The error bars represent the standard deviation; in the same year, statistical significance between the main season and the ratoon season is indicated by different lowercase English letters using Student’s *t*-test (*p* < 0.05).

A holistic assessment of RR must consider the yield-scaled global warming potential (GWP). Evidence suggests that while total seasonal emissions from a ratoon system may be significant, the higher total grain output can lead to a lower yield-scaled GWP—meaning less GHG emitted per ton of rice produced—enhancing its climate efficiency compared to some single-crop systems [36]. For example, Song et al. [54] reported that the ratoon season alone exhibited significantly lower GWP compared with the main crop during the period of 2017–2019 (Figure 2a). However, the GHGI (greenhouse gas intensity, defined as the yield-corrected GWP) for the follow-up season was higher but showed no significant difference (Figure 2b), which is attributable to its lower yield compared to the main crop. It is important to note that these comparisons refer to the individual ratoon season versus the main crop season, not to the complete main–ratoon system versus single-season cropping. Thus, raising the yield level of ratooning rice becomes crucial in reducing the yield-scaled GWP. According to a meta-analysis of 571 paired measurements from 32 publications, the shift to RR from either SR or DR systems significantly lowered GHGI by 7.9% and 17.3%, respectively, primarily due to enhanced grain yield (Table 1). Notably, an optimized cultivation for RR (oRR), defined as the integration of practices like alternate wetting and drying (AWD) irrigation, refined nitrogen management, and high-yielding variety selection, further reduced GHGI by 18.8% relative to standard RR practices [55]. Ultimately, the net GHG balance of an RR system is not predetermined but is a function of management decisions.

### 3.2. Ratoon Crop in Dryland

#### 3.2.1. Key Differences in GHG Emissions Between Dryland and Wetland Ratoon Crops

Ratoon crops such as cassava (*Manihot esculenta*), cotton (*Gossypium* spp.), garlic chives (*Allium tuberosum* Rottler ex Sprengle), sorghum (*Sorghum bicolor* [L.] Moench), pigeonpea (*Cajanus cajan* [L.] Millsp.), and sugarcane (*Saccharum officinarum* L.) are usually grown in dryland systems. Several key factors distinguish the GHG emission profiles of dryland ratoon crops from those of paddy rice (Table 2).

#### 3.2.2. GHG Emissions and Mitigation Mechanisms for Ratooning Crops in Dryland

The principles of ratooning apply to several other crops, each with distinct GHG emission profiles and mitigation levers. Ratoon crops in dryland systems offer credible and quantifiable GHG mitigation benefits, although the evidence base varies significantly by crop. 

Ratoon sugarcane stands out as a model system where the shift away from burning to green-cane trash-blanketing (GCTB), coupled with reduced tillage, has demonstrably lowered the carbon footprint and turned plantations into net carbon sinks in many cases. A study in São Paulo, Brazil, quantified this shift, showing a reduction in field emissions from approximately 2300 kg CO_2_e ha^−1^ to 2100 kg CO_2_e ha^−1^ when mechanical harvesting replaced burning between 2006 and 2012 [56]. A holistic life-cycle assessment of ratoon sugarcane systems demonstrated superior sustainability over plant cane cultivation, with significantly lower energy demand, reduced environmental impacts, and enhanced economic efficiency [57].

For sorghum, the potential for significant soil carbon sequestration is high due to its C4 physiology and extensive root system, but more field-based GHG flux measurements from ratoon systems are needed to move beyond model-based estimates [58,59].

Moreover, pigeon pea, as a perennial legume, offers a powerful dual mitigation mechanism: it avoids substantial GHG emissions by negating the need for N-fertilizer through biological nitrogen fixation (BNF) and actively sequesters significant amounts of carbon in the soil [60,61]. However, it should be noted that consolidated quantitative data on soil gas fluxes (kg CO_2_ ha^−^^1^ yr^−^^1^) for ratoon sorghum, cotton, and pigeon pea remain limited. Most available data are derived from model-based estimates or single-site studies, and field-scale, multi-year GHG flux measurements are needed to validate these predictions. Future research should prioritize field-scale, multi-year GHG flux measurements for ratoon sorghum, cotton, and pigeon pea to validate model predictions and provide the robust data necessary for developing climate-smart agricultural policies in vulnerable dryland regions.

## 4. Environmental Factors and Physiological Processes in Different Cropping Systems Influencing GHG Emissions

Environmental factors and physiological processes play a crucial role in the emissions of GHGs in cropping systems. Understanding the complex interactions between soil conditions, temperature, humidity, and physiology of different crops is essential for effectively mitigating these emissions.

### 4.1. Non-Ratoon Cropping System

Several environmental factors contribute to GHG emissions in cropping systems. One significant factor is soil condition. The type, texture, chemical composition, temperature, humidity, pH, redox potential, and organic matter content of the soil affect microbial activity, which in turn influences GHG emissions [62,63]. Additionally, temperature, rainfall, and other weather patterns also impact the release of GHGs from the soil [64]. A warm/humid climate leads to more carbon dioxide (CO_2_) emissions, while a relatively cold/dry climate and soil factors result in more nitrous oxide (N_2_O) emissions [65]. 

Furthermore, physiological processes in cropping systems greatly influence GHG emissions. One important process is the decomposition of organic matter, which releases CO_2_ into the atmosphere. This decomposition is driven by microbial activity and is influenced by factors such as soil moisture and temperature [66,67]. Additionally, the availability of nutrients and their uptake by plants also play a role in GHG emissions [29]. For example, excessive nitrogen fertilization can lead to the release of N_2_O [64]. Moreover, the N_2_O emissions from the soybean (*Glycine max*) plot were higher than those from the maize (*Zea mays*) plot [68].

### 4.2. Ratoon Cropping System

The ratoon cropping system presents several special aspects regarding environmental factors and physiological processes that influence GHG emissions.

One distinctive aspect is a prolonged duration of the cropping cycle, where the initial crop’s root system remains intact after harvest, allowing for the regrowth of subsequent crops from the ratoon shoots [8]. This extended cropping period impacts soil carbon dynamics [69] and microbial activity [70]. The presence of residual roots provides organic matter inputs to the soil, potentially influencing soil respiration rates [71] and increasing carbon sequestration rates [5]. 

Additionally, the ratoon cropping system alters nutrient cycling dynamics compared with traditional cropping systems. Nitrogen cycling in ratoon crops, particularly nitrogen fertilization practices, influences GHG emissions such as N_2_O [72]. Moreover, nutrient availability, particularly nitrogen, can fluctuate throughout the ratoon cycle due to the decomposition of previous crop residues and the release of stored nutrients from roots [5]. These fluctuations influence microbial processes involved in nitrogen transformations, such as nitrification and denitrification, which are key drivers of GHG emissions.

Furthermore, the presence of multiple roots at different ages within the same growing season can lead to variations in water and nutrient uptake patterns, affecting soil moisture levels and nutrient distribution [73]. These changes influence microbial activity and decomposition rates, subsequently impacting GHG emissions from soil [74].

Physiological processes within ratoon crops, such as shoot regrowth and root development, also play a role in GHG emissions. Root exudation, root turnover, and rhizosphere processes contribute to carbon and nutrient dynamics in the soil, influencing microbial activity and GHG production [70,72].

Overall, the ratoon cropping system presents a complex interplay of environmental factors and physiological processes that influence GHG emissions, highlighting the need for tailored management strategies to mitigate emissions while maximizing crop productivity.

## 5. Management of Ratoon Cropping for Reducing GHG Emission

**Figure 3:**
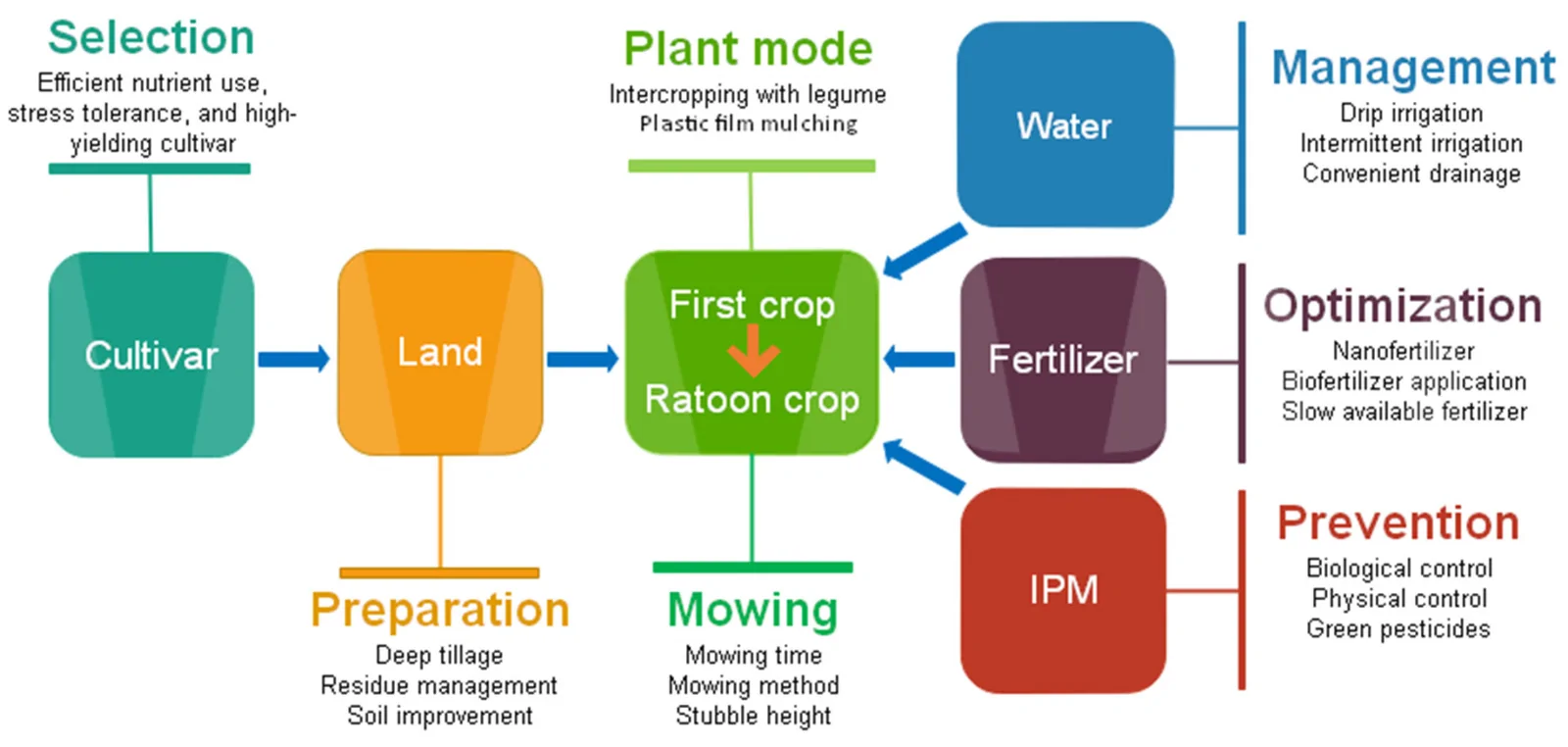
Workflow of ratoon cropping for reducing GHG emission. IPM, integrated pest management.

Wise crop management practices play a pivotal role in reducing GHG emissions within cropping systems [64]. Cultivar selection, land preparation, cultivation mode, mowing, optimized fertilizer application, integrated pest management (IPM), and water-saving irrigation [42,75,76,77,78] can help reduce the release of GHGs (Figure 3).

### 5.1. Cultivars Selection

Suitable cultivars reduce GHG emissions via core traits: (1) high biomass production (enhances carbon sequestration; net GWP per unit yield is comparable/lower despite increased biomass cycling) with high yield and quality [79,80,81]; (2) disease/pest resistance (reduces chemical pesticide use) [82]; (3) abiotic stress resilience (avoids additional inputs like irrigation/amendments); (4) slow residue decomposition (increases soil carbon storage); (5) efficient root architecture (traps soil carbon); (6) high fertilizer/water use efficiency (reduces N_2_O emissions and irrigation-related CO_2_); (7) optimized root exudates (suppresses methanogens) [83]. For example, suitable rice varieties reduce CH_4_ emissions by 20–50% [84,85,86]. 

**Figure 4:**
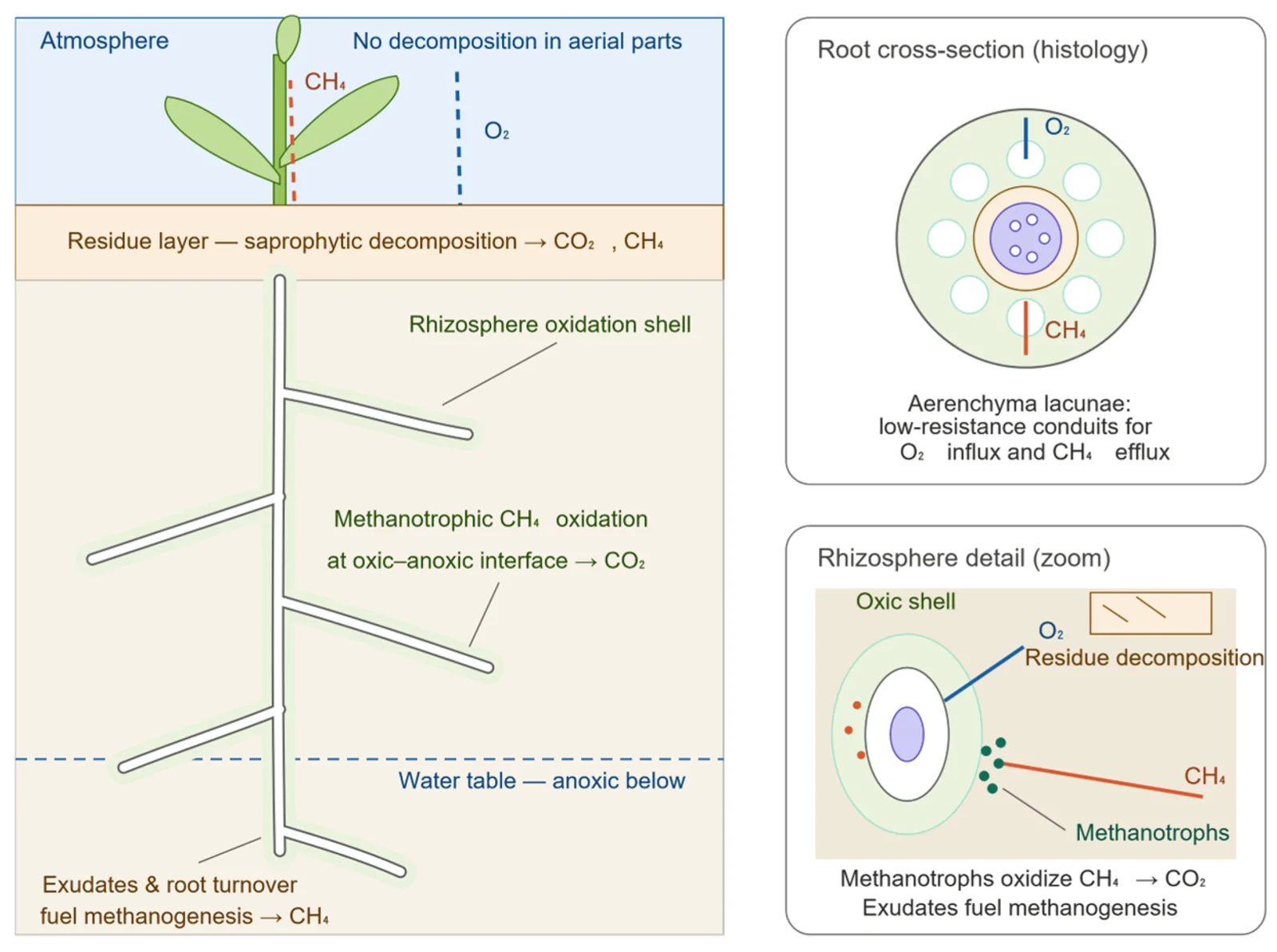
Histological and physiological determinants of trace-gas exchange in the ratoon crop root–soil system. The diagram locates residue decomposition and gas transport where they physically occur—at the soil surface and in the rhizosphere, rather than in the aerial plant parts, which serve solely as a photosynthetic organ and gas conduit (top left). Saprophytic decomposition of crop residues and stubble at the soil surface, together with root exudation and root turnover in anoxic bulk soil below the water table, supplies the substrates for methanogenesis (left panel). The histological cross-section (top right) reveals the aerenchyma lacunae—cortical air spaces that function as low-resistance conduits for downward O_2_ diffusion and upward CH_4_ efflux. Diffused O_2_ is radially leaked from the roots, generating a thin oxic shell that contrasts sharply with the anoxic bulk soil (bottom right). At this oxic–anoxic interface, methanotrophic bacteria oxidize a substantial fraction of the upward CH_4_ flux to CO_2_, attenuating net CH_4_ emissions; the remainder escapes to the atmosphere through the aerenchyma. This spatially explicit framework replaces the previous tree diagram and underpins the emission estimates presented in the subsequent sections.

Mechanistic understanding of why ratoon systems emit less CH_4_ per unit of grain than their single-crop counterparts begins with the spatial organization of decomposition and gas transport in the root–soil system (Figure 4). Decomposition is spatially segregated from the plant canopy: crop residues and stubble are mineralized saprophytically at the soil surface, whereas root exudation and root turnover supply labile carbon to methanogens in anoxic bulk soil below the water table. The plant itself acts as both conduit and filter. Aerenchyma lacunae in the root cortex provide low-resistance pathways through which O_2_ diffuses downwards while CH_4_ escapes upwards, bypassing the anoxic soil column. Critically, O_2_ leaked radially from the roots—particularly at the apex, where barriers to radial O_2_ loss are absent—sustains a thin oxic shell embedded within the anoxic matrix. At this oxic–anoxic interface, root-associated methanotrophs act as biological filters, oxidizing a substantial fraction of the potential CH_4_ flux to CO_2_ before it reaches the atmosphere. Ratoon cropping amplifies both sides of this balance: post-harvest stubble and residue inputs enrich methanogenic substrates, whereas the shallow, often intermittently aerated water regime of the ratoon season widens the oxic shell and strengthens methanotrophic suppression, contributing to the reduced greenhouse-gas intensity and net carbon-budget surplus observed under ratoon management [55,75]. The net emission therefore reflects this tension, quantified in the following section.

### 5.2. Land Preparation

Land preparation in a ratoon cropping system, which involves the cultivation of crops from the stubbles of previous harvests without replanting, can have significant impacts on GHG emissions. Analyzing from the perspectives of deep tillage and soil improvement offers insights into its environmental implications.

#### 5.2.1. Deep Tillage

Deep tillage (DT), a land preparation practice, involves the cultivation of soil at a depth greater than traditional tilling (<20 cm). This practice can influence GHG emissions in several ways: (1) Soil structure and aeration: DT can disrupt soil aggregates and increase soil aeration, which can stimulate the microbial oxidation of soil organic matter in the 0–20 cm soil layer [74]. Although deep cultivation increased the total CO_2_ emissions by 4.9–37.7% in wheat and maize cropping systems on loamy soils [87], it had no significant impact on N_2_O and CH_4_ emissions. (2) Soil carbon storage: A meta-analysis evaluating the response of soil organic carbon (SOC) storage to DT based on 430 comparative global data from 43 studies showed that DT can significantly increase SOC by 7.79% overall. Specifically, DT did not significantly promote SOC sequestration in the entire soil profile, but it significantly increased organic carbon in the 20–50 cm layer. In addition, the individual response of SOC to DT has high site specificity, and DT has greater benefits in soils in arid areas [88]. (3) Enhanced root penetration: It allows for greater root penetration and can enable crops to access nutrients from deeper soil strata, potentially enhancing plant growth and carbon uptake [89]. (4) Improved drainage: It can improve drainage, reducing the incidence of waterlogging, which in turn can mitigate methane emissions typically associated with anaerobic soil conditions [74].

In addition, it is important to recognize that DT is performed only once or rarely every 5 to 10 years. For such infrequent tillage events, the positive effects of transferring soil organic carbon deeper into the soil outweigh the possible negative effects of tillage [90]. However, this conclusion is based on long-term experimental data from temperate cropping systems and may not be directly generalizable to ratoon cropping systems, where the timing and frequency of soil disturbance may differ. The net increase across the assessed profile depends on the balance between deep transfer and surface losses, and should not be generalized without considering site-specific conditions.

#### 5.2.2. Soil Improvement

Soil improvement encompasses practices to enhance soil health and fertility, which can also impact GHG emissions: (1) Organic amendments: Incorporating organic amendments, like biochar and filter mud, can promote the growth of ratoon crops and soil quality by enriching stress-regulating metabolites and inhibiting pathogenic bacteria [91], potentially reducing GHG emissions by improving plant health [92,93]. According to reports, biochar application reduces emissions of N_2_O by 2.7–54% [94,95] and CH_4_ by 6–50% [96,97]. (2) Soil pH: Lime applications to correct soil acidity can also influence GHG emissions. Lime can increase soil pH, reducing N_2_O production by suppressing denitrifying bacteria [98,99,100]. (3) Moisture management: Soil improvement practices that enhance water retention can reduce the need for irrigation [101], thus indirectly reducing the energy-related CO_2_ emissions associated with water pumping. However, excessive moisture can lead to anaerobic conditions, potentially increasing CH_4_ emissions. Moreover, precise and targeted management approaches, such as utilizing gypsum and cover crops [102] or integrating alternate cropping sequences, have shown promising results in reducing GHG emissions [65,103]. 

### 5.3. Cultivation Modes

In a ratoon cropping system, cultivation modes particularly focusing on the practices of rotation/intercropping with legumes (cover crops) and mulching can have a significant impact on the reduction in GHG emissions.

#### 5.3.1. Rotation/Intercropping with Cover Crops

Rotation/intercropping with cover crops such as legumes in a ratoon cropping system can significantly influence the reduction in GHG emissions through various mechanisms. Legume intercropping can contribute to GHG mitigation through the following mechanisms: (1) Nitrogen fixation: Legumes possess the unique capacity to fix atmospheric nitrogen via symbiotic bacteria (rhizobia) in their root nodules. The residual nitrogen can reduce the associated CO_2_ and N_2_O emissions from fertilizer production, transportation, and field application [104]. For instance, sugarcane rotation with legumes (e.g. soybean) enhanced biological N_2_-fixation by 35 kg N ha^−^^1^ yr^−^^1^, while intercropping with Phaseolus beans increased it by 25 kg N ha^−^^1^ yr^−^^1^ [105]. (2) Soil carbon sequestration: Legumes contribute to soil organic carbon pools through root biomass addition and leaf litter. Organic matter from legumes is incorporated into the soil, enhancing its carbon sequestration capability [106]. This process offsets CO_2_ emissions and enhances soil health, potentially reducing the need for mechanical soil cultivation, which is a common source of CO_2_ emissions due to fossil fuel combustion in agricultural machinery [107]. (3) Improved soil structure: The root systems of legumes can contribute to better soil structure, leading to improved aeration and reduced soil compaction. Healthier soils with good structure favor microbial activity that can further enhance carbon sequestration [108] and reduce GHG emissions indirectly. (4) Efficient use of resources: Intercropping with legumes can lead to more efficient use of resources such as light, water, and nutrients [109]. Higher efficiency reduces the need for inputs like irrigation [110] and additional herbicides [102], both of which are linked to indirect GHG emissions, especially when the energy for these inputs is derived from fossil fuels. (5) Disease and pest management: Legumes can play a role in suppressing certain pests and diseases, potentially reducing the need for chemical pesticides [111]. The production, transportation, and application of pesticides are associated with GHG emissions, so their reduction is beneficial for mitigating climate change. (6) Crop diversification and resilience: Intercropping with legumes increases biodiversity within the cropping system, which can enhance ecosystem resilience to extreme weather events and reduce the likelihood of complete crop failure [112]. It has been shown that enhancing plant–soil–microbe interactions can boost agroecosystem resilience by 15–40% [113]. This diversification can lead to more stable carbon sequestration rates and avoid potential spikes in GHG emissions that occur when crops fail and require additional resource-intensive management practices to reestablish [114].

#### 5.3.2. Mulching

The use of mulching, such as plastic film, biodegradable film, and trash in ratoon cropping systems, offers several beneficial effects on GHG emissions reduction. The positive impacts of mulching on GHG mitigation include the following: (1) Moisture conservation and reduced irrigation needs: Mulching decreases soil evaporation, thereby conserving water and maintaining optimal soil moisture levels [110]. This reduction in the need for irrigation translates to lower energy use for water pumping, especially in regions where irrigation systems are powered by fossil fuels. Consequently, this decreased energy consumption leads to a reduction in CO_2_ emissions associated with energy production. (2) Soil temperature regulation: By modifying the microclimate around the plant roots, mulching can increase soil temperature in cooler climates, aiding in extending the growing season [110,115]. This can accelerate plant growth and development, leading to earlier maturation with reduced time in the field. A shorter crop cycle can lead to reduced tractor use and other field operations, thereby lowering fuel consumption and associated GHG emissions. (3) Weed suppression: The mulch acts as a physical barrier to weed germination and growth [116]. This suppression of weeds reduces the need for frequent tillage or herbicide applications, both of which are associated with CO_2_ emissions—tillage due to fuel use, and herbicides due to their production and transportation. (4) Enhanced fertilizer efficiency: By reducing nutrient losses through leaching or volatilization, mulching can increase the efficiency of fertilizer use [117,118]. Efficient use of fertilizer can lead to a reduction in the amount of fertilizer required, thereby decreasing the emissions associated with their production and application, particularly nitrous oxide, which is a potent GHG. (5) Reduction in methane emissions: In paddy fields under rice ratoon cropping, plastic mulching can help reduce methane emissions under non-continuously-flooded, water-saving management: by reducing percolation and moderating soil temperature, mulching keeps the soil largely unsaturated, thereby shortening the duration of anoxic phases in which obligate anaerobic methanogens produce CH_4_, while the longer aerobic phases favor methanotrophic CH_4_ oxidation [37,119,120]. This effect is conditional, however: if mulching is combined with continuously flooded and warm soil, elevated temperature would be expected to stimulate methanogenesis instead, and the accompanying oxic pulses may increase N_2_O emissions. In reference [121], all three treatments—plastic mulching (PM), straw mulching (SM), and no mulching (NM)—were managed with reduced irrigation, and the reported values are all expressed relative to continuous flooding: CH_4_ emissions declined by 68.8%, 61.4%, and 57.2%; N_2_O emissions rose by 84.8%, 89.1%, and 96.6%; GWP decreased by 50.7%, 47.5%, and 46.8%; and water use efficiency increased by 50.2%, 40.9%, and 34.0% [121]. Because these percentages do not isolate the effect of mulch, the additional effect of plastic mulching can be estimated only by comparing PM with NM under the same reduced irrigation regime, which corresponds to a further CH_4_ reduction of approximately 27% (0.312 vs. 0.428 of the continuous-flooding level). Direct mulched-versus-unmulched comparisons under identical water management remain scarce; therefore, these findings should be interpreted as effects of the respective management regimes rather than as an isolated mulching effect. (6) Increased carbon sequestration: Healthy plant growth promoted by mulching can lead to higher biomass production in the semi–arid area, which translates to greater carbon storage within the plant tissue [122].

While these points highlight the positive impacts of plastic film mulching on GHG emissions, it is important to also consider the environmental impact of the plastic waste generated. To address this, the use of biodegradable film mulch has been promoted as a sustainable alternative, offering the benefits of traditional plastic mulching without the long-term environmental pollution concerns [115].

### 5.4. Water Management

**Figure 5:**
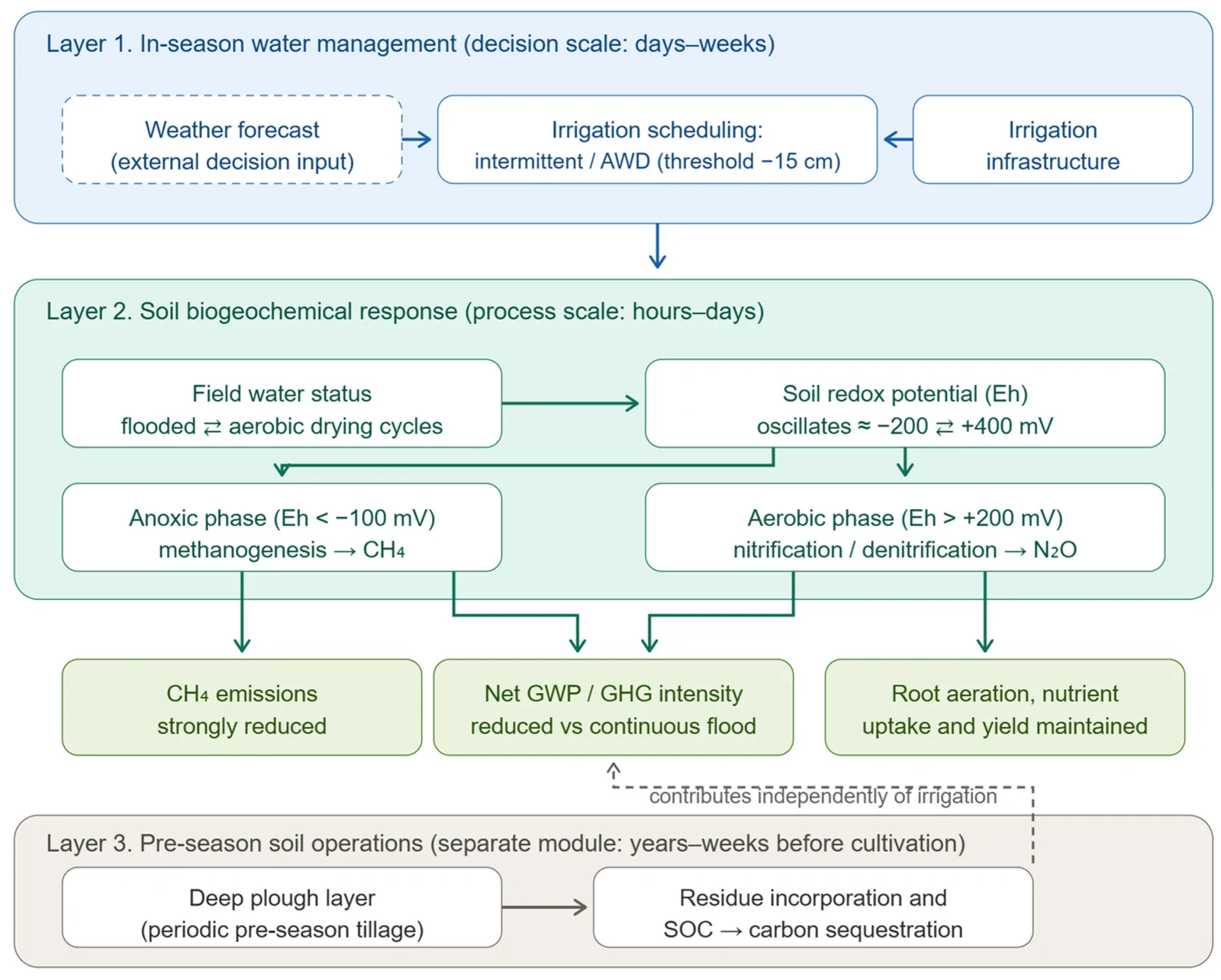
Mechanistic framework linking water management to greenhouse gas mitigation in ratoon cropping systems. Unlike conventional input–output schematics, the framework is organized around the causal chain that governs trace-gas fluxes: in-season irrigation decisions (Layer 1) regulate field water status, which drives the oscillation of soil redox potential (Eh) between anoxic (Eh < −100 mV) and aerobic (Eh > +200 mV) phases (Layer 2). Anoxic phases promote microbial methanogenesis and thus CH_4_ production, whereas aerobic phases stimulate nitrification and denitrification, generating pulsed N_2_O emissions. Under alternate wetting and drying (AWD) with a safe water table threshold of −15 cm, the suppression of methanogenesis during drying phases outweighs the moderate increase in N_2_O pulses, yielding a net reduction in global warming potential (GWP) and greenhouse gas intensity while maintaining root aeration, nutrient uptake and grain yield. Weather forecasts and irrigation infrastructure are shown as external decision inputs rather than biogeochemical drivers. Pre-season mechanical soil operations, including deep ploughing and residue incorporation, are depicted as an independent module (Layer 3) that contributes to soil organic carbon (SOC) accumulation independently of in-season water management. Dashed arrows denote influences that are not part of the water-mediated causal pathway.

Water management practices, including irrigation and drainage, affect soil redox conditions and microbial activity, thereby influencing GHG emissions such as CH_4_ and N_2_O in ratoon cropping systems. Proper management of irrigation and drainage can lead to significant reductions in GHG outputs from agricultural practices. The mitigation potential of ratoon systems cannot be inferred from a simple inventory of management practices; it emerges from the coupling of water regime and soil redox dynamics (Figure 5). Intermittent irrigation governed by safe AWD thresholds repeatedly lifts soil Eh above the methanogenic threshold (approximately −100 mV), interrupting the strictly anaerobic conditions under which methanogens convert acetate and H_2_/CO_2_ to CH_4_. Each re-flooding event, in turn, re-establishes anoxia and triggers denitrification, so the frequency and duration of drying cycles determine the trade-off between CH_4_ suppression and N_2_O pulses—a trade-off (Figure 5, Layer 2). By contrast, deep ploughing and residue incorporation operate on a different temporal scale altogether: they are mechanical interventions executed weeks to years before cultivation, and their influence on SOC stocks is largely decoupled from in-season water decisions. Distinguishing these three layers—decision, process and legacy management—is essential, because conflating them obscures the levers through which emissions are actually controlled. The following analysis outlines the impacts of irrigation and drainage on GHG emissions. 

#### 5.4.1. Irrigation Efficiency and Timing

Efficient irrigation systems, such as drip or subsurface irrigation, can reduce the amount of water needed, which decreases energy use for water pumping, thus cutting down CO_2_ emissions associated with energy consumption [123]. A comparative study demonstrated that drip irrigation reduced water consumption by 60%, energy use by 19.47%, and carbon emissions by 45% relative to traditional flood irrigation [124]. Moreover, the system of rice intensification (SRI)—a management approach integrating moisture control, which typically adopts saturated soil conditions rather than continuous flooding—has also been demonstrated to yield significant environmental benefits; specifically, one study showed that an SRI system reduced total GHG emissions by 80.1% compared to a conventional continuously flooded rice system [125]. The timing of irrigation also matters; for instance, avoiding unnecessary irrigation when the crop does not require it or when rain is forecast can prevent over-saturation of the soil, reducing the potential for GHG emissions [126,127].

#### 5.4.2. Water Table Control

In ratoon crops like rice, which is traditionally grown in flooded conditions, managing the water table can significantly impact CH_4_ emissions. Intermittent irrigation, as opposed to continuous flooding, can curtail anaerobic conditions that foster methanogenic bacteria [128] and enhance inter-root soil nutrient cycling and growth-promoting associated bacterial populations [129]. One practice, known as AWD irrigation, reduces CH_4_ emissions by 52–77%, N_2_O emissions by 52–73%, and the overall GWP by 3–13% [130] without significantly affecting yields [128,131]. In addition, mid-season drainage (MD)—a single, extended drainage period during the rice’s vegetative stage—is a viable methane mitigation strategy that can reduce total seasonal CH_4_ emissions by 20–77% (average 52%), with the extent of reduction depending on the severity of soil drying during the drainage event [132].

#### 5.4.3. Nutrient Uptake Optimization

Properly managed irrigation helps optimize the uptake of nutrients by plants, reducing the need for high amounts of fertilizers [133,134], which are associated with high levels of GHG emissions during their production and application. In particular, nitrogen-based fertilizers can convert to N_2_O if over-applied or inefficiently used [135].

#### 5.4.4. Reduced Fertilizer Leaching and Runoff

Excessive irrigation can cause leaching of nitrogen into groundwater and surface water systems [136,137]. Efficient irrigation and good drainage practices can reduce this leaching, thereby reducing water pollution and the potential for N_2_O emissions [138,139]. Additionally, reduced runoff can prevent the loss of soil organic matter, which, when eroded, can lead to the release of CO_2_.

#### 5.4.5. Soil Carbon Sequestration

Proper drainage can improve soil structure and aeration, leading to enhanced soil carbon storage capacity. Oxygen-rich soils can promote the growth of aerobic soil microorganisms, which contribute to carbon stabilization through the formation of stable microbial by-products (e.g., necromass) that associate with mineral surfaces [126,128]. However, it should be noted that the claim that aerobic microorganisms sequester carbon “more efficiently” than anaerobic microorganisms requires qualification. While aerobic conditions favor the production of recalcitrant microbial compounds that can persist in soil, anaerobic conditions can also promote carbon preservation through reduced decomposition rates and the formation of reduced organic matter complexes. The net effect on SOC stocks depends on the balance between input rates and decomposition kinetics under specific environmental conditions. Changes in microbial activity and carbon turnover rates should be distinguished from net SOC-stock accumulation, and the comparison between aerobic and anaerobic microbial efficiency should be interpreted within the context of specific ecosystems and management practices.

### 5.5. Optimized Fertilization

In ratoon cropping systems, optimizing fertilization is crucial to reduce GHG emissions. The production and application of fertilizers are significant sources of GHGs, particularly N_2_O, a potent GHG with a GWP many times that of CO_2_. The types of fertilizers used, and the manner in which they are applied, can significantly influence the magnitude of these emissions. Below, we summarize fertilizer types and application practices that can help reduce GHG emissions (Table 3). 

#### 5.5.1. Fertilizer Types for Reducing GHG Emissions

The following types of fertilizer can be employed to achieve this goal of reducing GHG emissions in ratoon cropping. In particular, urea substitutes offer a promising solution for reducing GHG emissions in agriculture by providing efficient nitrogen fertilization with reduced environmental impact [99]. These alternatives to traditional urea fertilizers aim to mitigate nitrogen-related emissions, particularly N_2_O, while maintaining or enhancing crop productivity. 

##### Enhanced Efficiency Fertilizers (EEFs)

EEFs are a broad category that includes controlled-release (slow-release) fertilizer, fertilizers with inhibitors (nitrification and urease inhibitors), and other products designed to increase nutrient use efficiency of crops. By improving efficiency, EEFs help to reduce the nutrient losses to the environment, including GHG emissions. For example, polymer-coated urea is a type of controlled-release fertilizer that has shown effectiveness in reducing N_2_O emissions in rice cropping systems [140]. A comprehensive meta-analysis of studies in rice systems revealed that the use of various EEFs resulted in an average N_2_O emission reduction of 28% and an average CH_4_ reduction of 15% compared to conventional urea [150].

##### Organic Fertilizers

Organic fertilizers, including compost, manure, and biochar, can improve soil health and increase carbon sequestration in the soil, potentially offsetting GHG emissions [144,151]. They usually release nutrients more slowly than synthetic fertilizers, reducing leaching and gaseous losses. While they can be beneficial, it is essential to manage their application carefully to avoid excessive nutrient application and subsequent GHG emissions. Substituting chemical fertilizers with fresh organic manure, fermented organic amendments, or their combined application reduced CH_4_ emissions from rice paddies by 19–59%, demonstrating the critical role of organic matter stability in methane mitigation [152].

##### Biofertilizers

Biofertilizers consist of living microorganisms that enhance plant nutrient uptake through various mechanisms, including nitrogen-fixing bacteria (e.g. *Azospirillum*), phosphate solubilization, and the production of plant growth-promoting substances [145]. By potentially reducing the need for synthetic nitrogen and phosphorus fertilizers, biofertilizers can lower GHG emissions.

##### Nanofertilizers

Nanofertilizers represent a promising avenue for reducing greenhouse gas (GHG) emissions in agriculture through enhanced nutrient efficiency and targeted delivery mechanisms [146]. These nanoscale materials, typically ranging from 1 to 100 nanometers in size, offer several advantages such as enhanced nutrient use efficiency, reduced environmental contamination, improved soil health, customized nutrient formulations, enhanced plant growth and yield, and longer shelf life and stability for optimizing nutrient uptake by crops while reducing environmental losses [153]. However, it is important to note that the widespread adoption of nanofertilizers requires thorough research to assess their long-term environmental impacts, including their potential to accumulate in soil and water systems [154]. Regulatory frameworks should be established to ensure the safe and responsible use of nanotechnology in agriculture, considering both agronomic benefits and environmental concerns.

#### 5.5.2. Fertilizer Application Methods for Reducing GHG Emissions

Beyond the choice of fertilizer, the method of application–"how, where, and when"–is equally critical for minimizing GHG losses. The overarching principle is to synchronize nutrient supply with crop demand as closely as possible.

##### Precision and Site-Specific Fertilization

These modern approaches move away from uniform, whole-field fertilizer applications. Using tools like GPS, soil sensors, drone imagery, and yield mapping, farmers can create detailed management zones within a field. Fertilizer is then applied at variable rates according to the specific needs of each zone, based on spatial data on soil and crop conditions [147]. By applying fertilizers only where they are needed, variable rate technology (VRT) can cut down on the overall use of fertilizers and the associated GHG emissions [149]. This reduces the excess use of fertilizers, especially nitrogen-based ones, which can produce N_2_O when over-applied. 

##### Split Fertilizer Application

Split fertilization can match plant uptake more closely than a single large application [148]. This tactic reduces the potential for nutrient losses to the environment, thereby reducing GHG emissions. 

##### Fertilizer Banding and Deep Placement

The physical placement of fertilizer within the soil profile is another powerful mitigation tool. Broadcasting fertilizer onto the soil surface, especially in flooded rice, exposes it to the aerobic soil-water interface, an ideal environment for nitrification and subsequent N_2_O loss. In contrast, placing fertilizer in bands at the time of sowing or injecting it in deep placement close to the plant roots can enhance nutrient uptake efficiency [135,155]. This technique can reduce the fertilizer rates required and reduce the potential for GHG emissions [156].

### 5.6. Integrated Pest Management

IPM in ratoon cropping systems can incorporate several specific measures to reduce GHG emissions. Generally, pest populations should be monitored regularly, and treatments should be applied only when pests exceed economic threshold levels. This ensures that interventions are timely and efficient, preventing unnecessary applications and reducing the carbon footprint associated with pest management. When it is necessary to control pests, biological, physical, and chemical control measures (Table 4) can be taken based on the type and quantity of pests. It should be noted that, if biological or physical methods can control pests, do not use chemical control, especially prohibited pesticides or chemicals.

#### 5.6.1. Biological Control

The most economical and environmentally friendly biological control is planting pest-resistant varieties [157]. An 89.52% reduction in the total environmental impact of pesticides was observed in Bt cotton compared to conventional varieties, attributable to the minimized use of chemical insecticides for bollworm control [170]. Secondly, beneficial organisms (e.g., predators, parasitoids, and pathogens) that naturally manage pest populations should be introduced [158,159,160]. This method reduces the need for chemical pesticides, which have a high carbon footprint due to their manufacturing, transportation, and application processes. Furthermore, RNA interference (RNAi), triggered by double-stranded RNA (dsRNA), has developed into a promising strategy for species-specific insect control [161]. In addition, cropping integrated with animals such as RR-duck co-culture significantly decreases GWP by 19–25% and maintains rice grain yield [162].

#### 5.6.2. Physical Control

Physical methods provide low-emission alternatives to synthetic pesticides. Physical control utilizes traps [163,164], barriers [165], mulches [166], and other mechanical methods to control pests [64]. For example, light trapping systems capture major rice pests including stem borers, leaf rollers, and plant hoppers, reducing insect populations by >30% within effective radii of 20–50 m [171]. These methods are often less GHG-intensive compared to the production and use of synthetic pesticides.

#### 5.6.3. Chemical Control

When chemical interventions become necessary, precision application strategies minimize environmental impacts. Precision application strategies—such as soil fumigation [167], nano-pesticide application [168], and multifunctional pesticide application (e.g., promoting ratoon ability and maturity) [169]—reduce the amount of pesticide required, and thus the energy and materials used in its production and application, thereby lowering the overall carbon footprint of pest management.

## 6. Discussion

### 6.1. Trade-Offs in Ratoon Cropping Systems

Ratoon cropping mitigates GHGs via reduced inputs and enhanced carbon sequestration, but faces trade-offs: (1) unoptimized nitrogen management may increase N_2_O emissions despite lower CH_4_ (shorter growth periods) [4]; (2) lower yield in the ratoon season than in the main season cropping can raise yield-scaled GHGI [54]. Mitigation requires balanced nutrient management and yield improvement via genetic breeding/optimized practices [55].

### 6.2. Cases Where Measures May Not Work Well

Despite the potential benefits of ratoon cropping, certain measures may not always be effective. For example, in regions with high soil acidity, the application of lime to correct pH levels can reduce N_2_O emissions by suppressing denitrifying bacteria [100]. However, the effect of soil pH changes on the specific activity of the N_2_O reductase enzyme (NosZ, encoded by the *nosZ* gene) deserves deeper consideration. NosZ is the terminal enzyme in the denitrification pathway that catalyzes the reduction of N_2_O to inert N_2_ gas. Its activity is strongly pH-dependent, with optimal function occurring near neutral pH (6.5–7.5) and significant suppression under acidic conditions (pH < 5.5). Lime application that raises soil pH toward neutral can therefore enhance NosZ activity, promoting complete denitrification to N_2_ and reducing the relative emission of N_2_O. Conversely, in soils that are already neutral or alkaline, lime application may not enhance NosZ activity and could potentially disrupt microbial community balance. Furthermore, lime-induced changes in soil pH can affect copper availability, which is a critical cofactor for NosZ function; reduced copper bioavailability at higher pH levels may partially offset the benefits of pH adjustment on NosZ activity. In areas with neutral or alkaline soils, lime application may have little to no effect on GHG emissions and could even lead to increased costs without substantial environmental benefits [99]. This underscores the importance of context-specific management strategies to ensure the effectiveness of GHG mitigation measures.

Similarly, while intermittent irrigation has been shown to reduce CH_4_ emissions in rice ratoon systems [76], it may not be suitable for regions with unreliable water supplies or where labor constraints limit the feasibility of precise water management practices [123]. In such cases, alternative water-saving techniques or crop varieties that are more tolerant to water stress may be more appropriate [172].

Beyond the environmental and agronomic dimensions discussed above, the widespread adoption of ratoon cropping systems faces significant economic hurdles that must be addressed. First, the high cost of specialized harvesters adapted for ratoon crops represents a substantial capital investment that may be prohibitive for smallholder farmers, particularly in developing countries where ratoon cropping is most prevalent. Second, ratoon harvests often exhibit reduced grain quality compared to main crop harvests, including lower grain weight, increased breakage rates, and variable milling quality, which can affect market prices and farmer income. Third, the repeated use of the same field without full replanting can lead to the accumulation of soil-borne pathogens and pest pressures over successive ratoon cycles, potentially increasing pesticide expenditures. Fourth, the labor requirements for ratoon management—particularly stubble height control during harvest and targeted fertilizer application—may differ from conventional systems and require additional training. These economic barriers underscore the need for context-specific policy support, including subsidized machinery programs, quality grading standards for ratoon grain, and integrated pest management strategies tailored to ratoon systems, to ensure that GHG mitigation benefits are realized without compromising farmer livelihoods.

### 6.3. Regional Limits and Context-Specific Strategies

The effectiveness of GHG mitigation measures in ratoon cropping systems is highly dependent on regional conditions. For instance, in tropical and subtropical regions where ratoon cropping is traditionally practiced, the warm and humid climate can accelerate soil organic matter decomposition, leading to higher CO_2_ emissions [63]. In contrast, in temperate regions, cooler temperatures may slow down microbial activity, reducing CO_2_ emissions but potentially increasing N_2_O emissions due to altered nitrogen cycling [64].

Furthermore, the adoption of cover crops to enhance soil carbon sequestration may be more effective in regions with sufficient rainfall to support their growth [109]. In arid and semi-arid regions, the lack of water may limit the success of cover crops, necessitating alternative soil improvement practices such as the application of organic amendments like biochar [159]. Looking forward, it is essential to incorporate simulations of global warming scenarios and extreme heat or water stress into the assessment of ratoon cropping sustainability. Climate model projections indicate that rising temperatures and increased frequency of extreme weather events (droughts, floods, heatwaves) could significantly affect the regrowth rate of basal buds in ratoon crops, potentially reducing ratoon yield stability. Under elevated CO_2_ and temperature scenarios, soil microbial activity may accelerate, potentially increasing decomposition rates and reducing the net SOC sequestration benefits of ratoon systems. Conversely, extended growing seasons in some temperate regions could enhance ratoon crop productivity. These climate crop GHG feedbacks represent a critical research frontier that should be addressed through integrated climate-crop modeling studies combined with long-term field observations across diverse agroecological zones.

## 7. Conclusions

The utilization of the ratoon cropping system offers a promising option for counteracting GHG emissions and advancing carbon sequestration. Comprehensive and informed management of the ratoon cropping systems in relation to climatic factors and physiological processes is imperative for the successful mitigation of GHG emissions. Implementing proper soil management, including conservation tillage and organic matter incorporation, can help reduce GHG emissions. Moreover, optimizing nutrient management practices, such as employing precision fertilization techniques, can minimize nitrogen losses and consequently reduce N_2_O emissions. In addition, by developing and implementing crop genetic improvement strategies, such as breeding for enhanced photosynthetic efficiency, stress tolerance, and nutrient uptake efficiency, the overall GHG emissions from ratoon cropping systems can be effectively reduced. The integration of climate-resilient and high-yielding crop varieties will play a pivotal role in achieving sustainable intensification of ratoon cropping systems. Furthermore, offering education and extension services to farmers regarding IPM practices can result in more efficient pest management strategies, leading to reduced GHG emissions. Knowledge dissemination about sustainable practices can enhance the adoption of techniques that decrease reliance on high-GHG-emitting inputs.

In conclusion, this review article provides a comprehensive overview of the methods and techniques employed to mitigate GHG emissions in ratoon cropping systems. The findings underscore the potential of ratoon cropping as an environmentally sustainable agricultural practice. By analyzing specific interactions between ratoon crops and their environments, and implementing effective crop management strategies, it is possible to reduce GHG emissions; however, it is important to qualify the claim that ratoon cropping "significantly reduces GHG emissions." While ratoon systems can lower yield-scaled GHG intensity (GHGI) compared to single- or double-cropping systems, this benefit is contingent upon proper water management practices such as alternate wetting and drying (AWD). Without such management, continuously flooded ratoon systems may exhibit elevated CH_4_ emissions compared to properly managed single-crop systems. This contributes to global efforts in combating climate change and promotes more sustainable farming practices, enhancing both environmental health and agricultural productivity. Our findings aim to inform policymakers, researchers, and farmers alike on the potential pathways towards a more sustainable and environmentally friendly agricultural future. Furthermore, this review proposes practical guidelines for incorporating ratoon cropping into global carbon markets, including: (1) establishing standardized protocols for measuring and verifying SOC sequestration in ratoon systems over multi-year timeframes; (2) developing additionality benchmarks that account for baseline emission scenarios; (3) addressing permanence and leakage concerns through buffer pools and monitoring requirements; and (4) creating verification frameworks that integrate both soil carbon benefits and avoided emissions from reduced input use. Finally, it is important to note that reducing GHG emissions in ratoon cropping systems requires empirical verification of adaptation strategies, co-benefits, and potential trade-offs. While certain strategies may effectively reduce emissions, they might also impact other aspects of crop production, such as yield or water usage. Therefore, adopting a comprehensive approach is essential to achieve a harmonious balance between reducing emissions and facilitating sustainable agricultural practices.

## Figures and Tables

**Table 1:** Impacts of cultivation system conversions (SR-to-RR, DR-to-RR, and RR-to-oRR) on CH_4_ emissions, grain yield, GWP, and GHGI in rice ratooning systems.

Items	RR vs. SR		RR vs. DR		oRR vs. RR	
	*n*	Average Change %	*n*	Average Change %	*n*	Average Change %
CH_4_ emission	39	35.4	24	23.2	53	–19.7
Grain yield	39	30.6	24	7.4	60	0.6
GWP	39	43.3	23	30.0	41	–15.6
GHGI	39	–7.9	23	–17.3	41	–18.8

Note: RR, conventional ratoon rice fields; SR, single-season rice; DR, double-season rice; oRR, optimized cultivation for RR. *n* denotes the number of paired observations. GWP, global warming potential; GHGI, greenhouse gas intensity. Data in the table are derived from [55] without conducting a new secondary analysis.

**Table 2:** Key differences in GHG emissions between dryland and wetland ratoon crops.

Items	Ratoon Crop in Dryland	Ratoon Crop in Wetland
Water Management	Rainfall or limited irrigation	Mostly flooded conditions
Soil Conditions	Aerobic	Anaerobic
Main GHG emissions	N_2_O	CH_4_
Residue Management	Generally returning residues	Generally removing residues

Note: The residue-management options above are qualified by the outcome metric used for judgment (seasonal CH_4_ emissions vs. net SOC accumulation/net GHG balance), the water regime, and nitrogen availability; they are context-dependent rather than general rules for all dryland or wetland ratoon crops.

**Table 3:** Fertilization measures for reducing GHG emissions in ratoon cropping.

Fertilization	Specification	Applied Crop and References
**Chemical fertilizers**	Slow-release fertilizer	Rice [140]
	Nitrification inhibitors	Vegetable [141]
	Urease inhibitors	Maize [142]
	Silicon fertilizer	Rice [143]
	Organic Fertilizer	Rice [144]
	Biofertilizer application	Sugarcane [145]
	Nanofertilizer application	Rice [146]
**Organic manners**	Precision fertilization	Rice [147]
	Split fertilizer application	Rice [148]
	Site-specific fertilizer application	Rice and wheat [149]
	Integrated nutrient management	Rice [144]
	Fertilizer banding	Rice [135]

**Table 4:** Main measures of IPM for reducing GHG emissions in ratoon cropping.

Main Measures	Specific Method	Applied Crop and References
**Biological control**	Pest-resistant cultivar	Cotton [157]
	Natural enemy	Sugarcane [158]
	Biocontrol agents	Sugarcane [159]
	Pheromone trapping	Sugarcane [160]
	RNAi-based technology	Cotton [161]
	Integration with animals	Rice [162]
**Physical control**	Light trapping	Sugarcane [163]
	Sticky board trapping	Sugarcane [164]
	Pest barrier	Rice [165]
	Mulching	Potato [166]
	Soil drying by sunning	Rice [64]
**Chemical control**	Soil fumigation	Sugarcane [167]
	Nano-pesticide application	Tomato [168]
	Multifunctional pesticide	Sugarcane [169]

## Data Availability

No new experimental data were created or analyzed in this review. All data supporting the findings of this review are drawn from previously published studies cited in the reference list.
